# Sortase-Mediated Ligation of Purely Artificial Building Blocks

**DOI:** 10.3390/polym10020151

**Published:** 2018-02-06

**Authors:** Xiaolin Dai, Diana M. Mate, Ulrich Glebe, Tayebeh Mirzaei Garakani, Andrea Körner, Ulrich Schwaneberg, Alexander Böker

**Affiliations:** 1 Fraunhofer Institute for Applied Polymer Research IAP, Geiselbergstr. 69, 14476 Potsdam-Golm, Germany; xiaolin.dai@iap.fraunhofer.de (X.D.); ulrich.glebe@iap.fraunhofer.de (U.G.); 2 Lehrstuhl für Polymermaterialien und Polymertechnologie, University of Potsdam, 14476 Potsdam-Golm, Germany; 3 DWI—Leibniz Institute for Interactive Materials e.V., Forckenbeckstr. 50, 52056 Aachen, Germany; diana.mate@icp.csic.es (D.M.M.); t.mirzaeigarakani@biotec.rwth-aachen.de (T.M.G.); koerner@dwi.rwth-aachen.de (A.K.); 4 Institute of Biotechnology, RWTH Aachen University, Worringer Weg 3, 52074 Aachen, Germany

**Keywords:** sortase-mediated ligation, enzymes, block copolymers, nanoparticles

## Abstract

Sortase A (SrtA) from *Staphylococcus aureus* has been often used for ligating a protein with other natural or synthetic compounds in recent years. Here we show that SrtA-mediated ligation (SML) is universally applicable for the linkage of two purely artificial building blocks. Silica nanoparticles (NPs), poly(ethylene glycol) and poly(*N*-isopropyl acrylamide) are chosen as synthetic building blocks. As a proof of concept, NP–polymer, NP–NP, and polymer–polymer structures are formed by SrtA catalysis. Therefore, the building blocks are equipped with the recognition sequence needed for SrtA reaction—the conserved peptide LPETG—and a pentaglycine motif. The successful formation of the reaction products is shown by means of transmission electron microscopy (TEM), matrix assisted laser desorption ionization-time of flight mass spectrometry (MALDI-ToF MS), and dynamic light scattering (DLS). The sortase catalyzed linkage of artificial building blocks sets the stage for the development of a new approach to link synthetic structures in cases where their synthesis by established chemical methods is complicated.

## 1. Introduction

Sortases are enzymes that naturally occur in the cell wall of Gram-positive bacteria. As transpeptidases, sortases link distinct peptide sequences by forming peptide bonds. They are grouped into six different classes, namely sortases A to F. Enzymes of these six classes have diverse functions, most importantly anchoring a large number of functionally different proteins to the cell wall (classes A, B, D, and E) and pili assembly (class C) [1]. Among the sortase superfamily members, sortase A (SrtA) is the one that has been more deeply studied [2,3]. The catalytic mechanism involves the nucleophilic attack by the cysteine residue in the active site of the enzyme to the carbonyl carbon of threonine in the recognition sequence LPXTG (for class A, being X any amino acid). This leads to the cleavage of the amide bond between threonine and glycine and to the formation of a thioester-acyl intermediate [4]. This intermediate is then resolved by the nucleophilic attack of the amine group from an oligoglycine motif. Consequently, a peptide bond is formed between the two substrates and SrtA regenerated [4].

In recent years, the use of SrtA was investigated for specific coupling reactions in non-natural environment [5,6,7,8]. Sortase-mediated ligation (SML) proved to be well suited for the enzymatic linkage between two proteins [9,10,11], lipids and proteins [12], proteins and DNA [13], as well as polypeptides and sugars (neoglyco-conjugate) [14]. Moreover, proteins were labeled with small molecules like fluorescent dyes, polymerization initiators, and functional units for the formation of e.g., fusion proteins, antibody-drug conjugates, and dendrimers [15,16,17,18,19,20,21,22,23]. Furthermore, SrtA was applied for peptide cyclization [24], virus modification [25], labelling surfaces of living cells with proteins or small molecules [26], combining protein purification and site-specific bioconjugation [27,28] as well as the formation of protein–polymer conjugates [29,30]. Sortagging was also carried out with immobilized sortases [31,32]. In addition, assays for bioconjugation were developed with SrtA [33]. Finally, proteins were immobilized by sortase on different surfaces, such as polystyrene beads, nano- and microparticles, microgels, planar substrates, gold surfaces, or biosensor chips [34,35,36,37,38,39,40,41]. However, to the best of our knowledge, a universal study for the linkage of various completely artificial chemical building blocks is not reported yet.

**Scheme 1:**
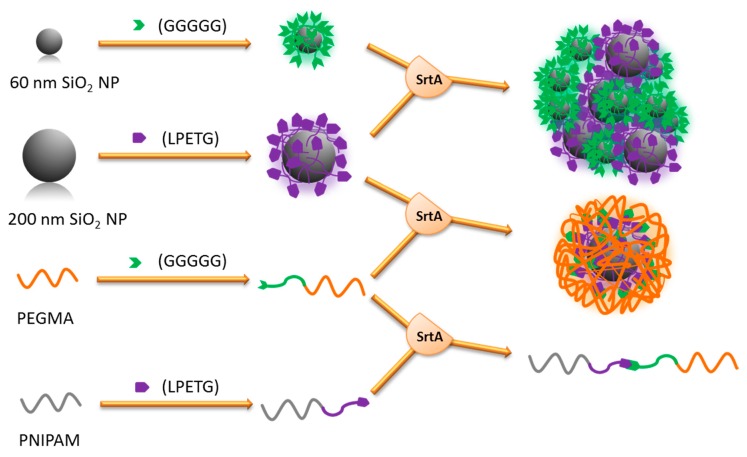
Strategy for the linkage of artificial building blocks by SrtA. The nanoparticle and polymer building blocks were functionalized with peptides containing the recognition sequence or nucleophile of SrtA and subsequently nanoparticle (NP)–NP, NP–polymer, and polymer–polymer structures formed by sortase-mediated ligation (SML).

Here, we demonstrate the linkage of entirely synthetic building blocks by SML. Beyond enzymatic approaches, SML is one of the most promising methods for the linkage of building blocks in general because of its high selectivity towards specific peptide motifs, the very robust nature of sortase, synthetic and commercial accessibility of all required compounds (including sortase), the simplicity to perform sortagging in a lab and possibilities to reach high yields [3,42]. Chemical linking technologies such as click chemical reactions are established nowadays [43,44], but are restricted to diverse functional chemical groups and the introduction of orthogonal peptide motifs may open new paths. This is even more relevant in light of the perspective that the term “bio-click chemistry” was already postulated as this enzymatic approach possesses many of the useful properties of click chemistry [38]. Therefore, we assume that sortase-mediated ligation technique could be promising for future use in materials science. As a proof of concept, we chose well established systems in chemistry, namely silica nanoparticles (NPs) of different sizes and poly(ethylene glycol) (PEG) as well as poly(*N*-isopropyl acrylamide) (PNIPAM). All building blocks were equipped with the respective peptide motifs for SrtA reaction at first. Afterwards, NP–polymer hybrids, NP–NP, and polymer–polymer structures were formed under SrtA catalysis (Scheme 1). We chose to link the peptides to nanoparticle surfaces and polymer functional end groups by thiol click reaction between cysteine residues and C=C-double bonds. Thus, we designed peptide motifs with LPETG or GGGGG sequences—at the C-terminus and N-terminus, respectively—and a cysteine residue on the opposing N-terminus or C-terminus, respectively. The peptides also contain 3–13 additional amino acids as spacers between recognition sequence and cysteine. This allowed to prevent steric hindrance during sortase catalysis when the peptides are linked to the building blocks. Longer spacer sequences were chosen for the NPs as we expected higher steric demand on the surface of the nanoparticles. Beyond that, the spacer sequences contain tryptophan and phenylalanine with aromatic residues that facilitate the analysis of the linkage of the peptides to the artificial building blocks by having characteristic bands in Raman spectra (see Supporting Information for exact peptide sequences).

## 2. Materials and Methods 

All chemicals used were of analytical reagent grade or higher quality, purchased from Sigma-Aldrich Chemie (Taufkirchen, Germany) or Alfa Aesar (Karlsruhe, Germany) if not stated otherwise. Poly(ethylene glycol) methyl ether acrylate (*M*_n_ = 2000 g/mol) and maleimide terminated poly(*N*-isopropyl acrylamide) (5500 g/mol) were used from Sigma-Aldrich. Peptides were received from Biotrend Chemikalien GmbH (Köln, Germany); the exact sequences of the four peptides used are listed in the synthetic procedures below. Protein ladder was purchased from ThermoFisher Scientific (Waltham, MA, USA) (PageRuler^TM^ Prestained Protein Ruler).

Transmission electron microscopy (TEM). TEM was performed on a Philips CM-200 device operating at 120 kV. The samples were deposited on nitrogen glow discharged carbon film coated grids, which were subsequently washed three times with Millipore water.

Matrix assisted laser desorption ionization mass spectrometry (MALDI-ToF MS). Spectra were acquired using a 337 nm laser Bruker microflex MALDI-ToF mass spectrometer (Bruker, Bremen, Germany) with pulsed ion extraction. The masses were determined in positive ion linear mode. The sample solutions were applied on a ground steel target using the dried droplet technique. Mass calibration was performed with external calibration. Polymer samples were prepared as follows: α-cyano-4-hydroxycinnamic acid (CCA) was used as matrix substance in a 10 mg/mL solution in Millipore water:acetonitrile 7:3 with 0.1% trifluoroacetic acid. Sodium trifluoroacetate was used as salt in 0.1 mol/L solution in the same solvent mixture. Sample (5 mg/mL), matrix and salt solutions were mixed in 5:20:1 ratio and 2 µL of the mixture applied on the target. The SrtA sample was prepared as follows: Super-DHB, a 9:1 mixture of 2,5-dihydroxybenzoic acid (DHB) and 2-hydroxy-5-methoxybenzoic acid, was used as matrix substance in a 50 mg/mL solution in Millipore water:acetonitrile 1:1 with 0.1% trifluoroacetic acid. The sample was applied on the target using ZipTip_C18_ pipette tips (Millipore, Darmstadt, Germany).

Raman spectroscopy. Raman spectra were measured with a Bruker RFS 100/S instrument (Bruker, Bremen, Germany) equipped with a 1064 nm Nd:YAG laser. The laser power was between 150–1000 mW and typically 1000 scans with spectral resolution of 4 cm^-1^ were used. The solids were measured in aluminium pans under 180° reflection geometry.

Dynamic light scattering (DLS). DLS measurements were performed on a Malvern (Worcestershire, UK) Zetasizer Nano ZS device at 20 °C. Assuming Mark–Houwink parameters to be A = 0.428 and B = 7.67 × 10^−5^, 173° backscattering was analyzed with equilibration time of 120 s. Multi-parameter analysis was performed on an average of three runs for every data point.

Field emission scanning electron microscopy (FESEM). FESEM measurements were performed on a Hitachi S4800 Field Emission SEM operated at 1–2 kV with a 10 mA current. The samples were prepared by placing a drop on a silicon wafer. The samples were air-dried before being placed into the specimen holder.

Expression and purification of SrtA. Sortase A from *Staphylococcus aureus* (SrtA) was expressed in genetically modified form without its transmembrane domain (SrtA_Δ59_) to favor soluble expression after cell lysis and with a His-tag at the N-terminus to facilitate the purification of the enzyme. The corresponding gene was cloned into the pET28a vector between the restriction sites NdeI and XhoI. Expression of SrtA_Δ59_ was performed in flask. In detail, a pre-culture of *E. coli* BL21 (DE3) harboring the pET28a-SrtA_Δ59_ plasmid (5 mL TB media, 100 μg/mL ampicillin) were inoculated from a glycerol stock and incubated (16 h, 37 °C, 250 rpm) in a Multitron II Infors shaker. The main culture (1 L flask, 200 mL TB media, 100 μg/mL ampicillin) was inoculated with 1 mL pre-culture and incubated for ~2 h at 37 °C and 250 rpm. At an OD_600_ of ~1 the main culture was induced with 0.2 mM isopropyl β-d-1-thiogalactopyranoside (IPTG) followed with incubation in the same shaker (16 h, 30 °C, 200 rpm). The expressed cells were harvested by centrifugation (30 min, 3220 *g*, 4 °C) (Eppendorf centrifuge 5810 R) and pellets were kept at −20 °C to favor further cell lysis. Cell pellets were resuspended (5 mL 50 mM Tris-HCl buffer pH 7.5 containing 0.8 mg/mL lysozyme) and incubated 45 min at 37 °C. After centrifugation (30 min at 12,000 *g* and 4 °C), the clear supernatant was filtered with a 0.20 µm filter (Millex-LG, Merck Millipore, Billerica, MA, USA). The enzyme was purified to homogeneity by Ni^2+^-affinity chromatography using a 5 mL HisTrap FF column (GE Healthcare, Little Chalfont, UK) connected to an ÄKTA*prime*. Binding buffer (buffer A) was 50 mM Tris-HCl buffer, 5 mM imidazole, pH 7.5, and elution buffer (buffer B) was Tris-HCl buffer, 500 mM imidazole, pH 7.5. A linear gradient from 10 mM imidazole (2% buffer B) to 300 mM (60% buffer B) was used for 150 min at a flow rate of 1 mL/min. Amicon ultra-15 centrifugal filter units with 10 kDa cut-off (Merck Millipore Ltd., Tullagreen, IRL) were used for preparation of concentrated sortase samples. The purity degree was checked by SDS-PAGE and MALDI-ToF mass spectrometry. SDS-PAGE samples were prepared by mixing 30 μL purified SrtA_Δ59_ and 10 μL 4× SDS loading buffer. The mixture was heated at 95 °C for 5 min. Eight microliters of the samples were loaded and analyzed in 12% acrylamide SDS-PAGE gels. As expected, the molecular mass of the protein was about 19 kDa, more specifically, 18,942 Da according to the MALDI-ToF mass spectrum (Figure S1).

Preparation of SiO_2_ nanoparticles and surface modification to obtain a C=C coating. For the preparation of 200 nm NPs, molar concentrations of Millipore water, NH_3_ (28–30% aqueous solution) and tetraethyl orthosilicate (TEOS) of 5, 0.2, and 0.2 mol/L, respectively, were used, while values of 1, 0.2, and 0.2 mol/L, respectively, were used for the preparation of 60 nm NPs. First, a mixture of ethanol, Millipore water, and ammonia was prepared to achieve the mentioned concentrations. After 30 min equilibration, TEOS was added. The experiments were conducted with 50 mL ethanol at room temperature for 24 h. For surface modification, 3-(trimethoxysilyl) propyl methacrylate (MPS) was directly added to the dispersion at room temperature. After 12 h stirring, the mixture was refluxed for 1 h to ensure the covalent bonding. The silica nanoparticles were then washed with ethanol by centrifugation and ultrasonication several times, until the supernatant did not show UV absorption from MPS anymore.

Functionalization of NPs with peptides. 200 nm NPs were reacted with peptide 1 (H-Cys-Ile-Arg-His-Met-Gly-Phe-Pro-Leu-Arg-Glu-Phe-Leu-Pro-Glu-Thr-Gly-OH), while 60 nm NPs were functionalized with peptide 2 (H-Gly-Gly-Gly-Gly-Gly-Phe-Glu-Arg-Leu-Pro-Trp-Phe-Trp-Gly-Met-His-Arg-Ile-Cys-OH). C=C-functionalized NPs and peptide were mixed in 1.1:1 molar ratio (peptide: C=C group) with 3 mol % 4,4′-azobis(4-cyanovaleric acid) (ABCVA) in water. The mixture was stirred in N_2_ atmosphere for 24 h under exposure to UV light (365 nm). Afterwards, the NPs were washed with Millipore water by centrifugation and ultrasonication.

Conjugation of peptides to polymers. PEGMA and maleimide terminated PNIPAM were reacted with peptide 3 (H-Gly-Gly-Gly-Gly-Gly-Trp-Phe-Trp-Cys-OH) and peptide 4 (H-Cys-Ile-Arg-His-Phe-Leu-Pro-Glu-Thr-Gly-OH), respectively. Polymer and peptide were mixed in equivalence ratio of 1.1:1 in PBS buffer (pH = 7.4) and stirred under N_2_ atmosphere for 24 h at RT. The obtained products were dialyzed two times against Millipore water using dialysis membrane with a MWCO of 1 kDa for 24 h.

SrtA reaction. The SrtA reactions between two substrates were conducted by the following procedure. A typical reaction mixture of 100 µL consisted of 30 µL aqueous solution of the two substrates with approximately 50-times excess of GGG substrate with respect to LPETG substrate, 20 µL SrtA (7.95 mg/mL), 20 µL 250 mM Tris-HCl and 750 mM NaCl (pH 7.5), 20 µL 25 mM CaCl_2_, 10 µL Millipore water, and was conducted at 28 °C (PNIPAM–PEG) or 37 °C (NP–PEG, NP–NP) in thermomixer for 24 h. NP–polymer samples were washed three times with Millipore water by centrifugation and ultrasonication.

## 3. Results

Silica nanoparticles were synthesized by sol-gel method with diameters of approximately 200 nm and 60 nm, respectively, similar to published procedures (Figure S2) [45,46]. Subsequently, the NPs were functionalized by reaction with 3-(trimethoxysilyl) propyl methacrylate (MPS) which provides accessible C=C-double bonds on the surface of the nanoparticles (Scheme S1 and Figure S3) [45,46]. Peptide 1 with LPETG recognition sequence was linked to the 200 nm NPs and peptide 2 with GGGGG to 60 nm NPs. The successful reaction was shown by Raman spectroscopy (Figure S4). As polymer blocks, we exemplarily chose two commercially available polymers with at least one defined end group providing ene functionality for reaction with the cysteine residues. Poly(ethylene glycol) methyl ether acrylate (PEGMA) with number average molecular mass (*M*_n_) of 2000 g/mol was equipped with the pentaglycine containing peptide 3. The second polymer, maleimide terminated poly(*N*-isopropyl acrylamide) with *M*_n_ of 5500 g/mol, was functionalized with LPETG containing peptide 4. The formation of the polymer–peptide conjugates was verified by analysis of their MALDI-ToF mass spectra (Figures S7 and S8).

### 3.1. Formation of Nanoparticle (NP)–Polymer Hybrids via Sortase-Mediated Ligation (SML)

**Figure 1:**
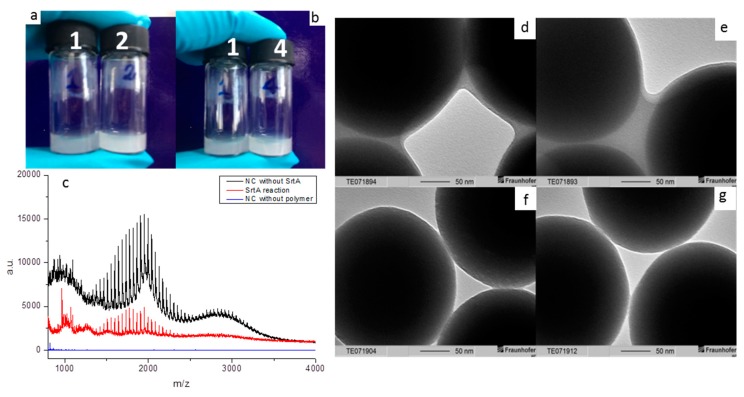
(**a**,**b**) Dispersability comparison between 1: Nanoparticle (NP)–polymer after SrtA reaction; 2: mixture of functionalized NPs and SrtA without polymer; 4: mixture of functionalized NPs and poly(ethylene glycol) (PEG) without SrtA; (**c**) MALDI-ToF mass spectra of NP–polymer sample after SrtA reaction showing only unmodified free polymer (red), negative control solution without SrtA showing both unmodified free polymer as well as peptide–polymer (black), and negative control with SrtA but without peptide–polymer (blue); (**d**–**g**) Transmission electron microscopy (TEM) images of NP–polymer hybrid particles after SrtA reaction (**d**,**e**), negative control without polymer (**f**), and negative control without SrtA (**g**).

SiO_2_ NPs of 200 nm in diameter, functionalized with the LPETG recognition motif, were reacted with an excess of GGGGG functionalized PEG under the catalysis of SrtA. After the two peptides were linked by sortase, a thin polymer shell was formed around the NPs. As PEG is very well water soluble, the NP–polymer hybrids dispersed much better in water than the NPs without polymer shell. Negative control samples were prepared in the meanwhile, which are mixtures without functionalized PEG or without SrtA, respectively. The dispersion of the NP–polymer structures is more transparent than the negative control samples of the same concentration, indirectly showing the successful linkage (Figure 1a,b). A quantitative UV–vis measurement confirmed the optical impression and verified that the sample of sortase reaction product is the most transparent one (Figure S12). Next, the supernatants after centrifugation from SrtA reaction and negative control samples were analyzed by MALDI-ToF mass spectrometry. The polymer–peptide conjugate (*m*/*z* ≈ 2800) can be detected in the solution of the negative control sample without SrtA (Figure 1c, black spectrum), but not in the SrtA reaction (Figure 1c, red spectrum). This implies that a high amount of polymer-peptide conjugate was linked to the NPs by SrtA. The polymer PEGMA (*m*/*z* ≈ 2000) was used in slight excess for the linkage with the peptide and is hence visible in the spectra of both the reaction and the negative control. Furthermore, the PEGMA starting material contains a series without methyl ether acrylate end group (Table S1) which therefore cannot be modified with peptide. The MALDI-ToF mass spectrum of the other negative control sample of NPs with SrtA but without polymer-peptide conjugate (Figure 1c, blue line) does not show anything in the low *m*/*z* range. Moreover, TEM images of NP–polymer particles show a big difference in comparison to the negative control samples (Figure 1d–g). The surface of the NPs is covered with a polymer film after linkage of the polymer while the negative control samples show a sharp edge of the NPs. The observed film is too thick to be caused by polymer–peptide of approximately 4000 g/mol. Therefore, we assume that free polymer is adsorbed to the covalently linked polymer. Interestingly, the thick polymer film stayed after three times washing of the NPs. In contrast, no free polymer can be observed around the NPs in the negative control without sortase after three times washing. Hence, it is expected that free polymer can only adsorb to NPs which are surface-modified with the polymer, but does not adsorb to non-functionalized or peptide-modified NPs. The same conclusion was taken from field emission scanning electron microscopy (FESEM) which showed a pronounced soft layer around the NPs after SrtA reaction (Figure S13).

### 3.2. Formation of NP–NP Clusters via SML

**Figure 2:**
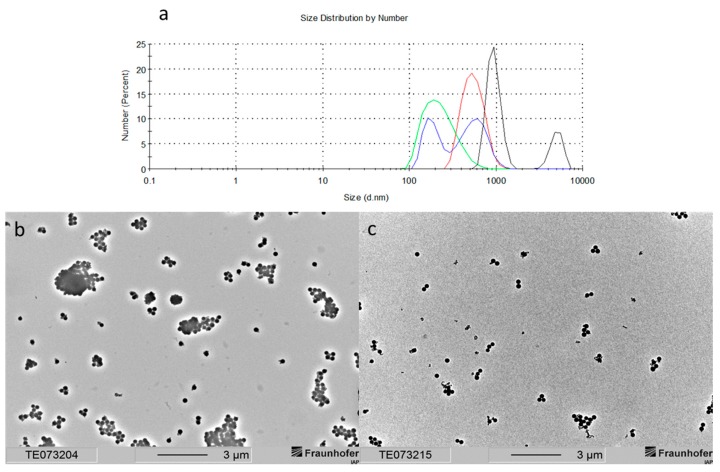
(**a**) Dynamic light scattering (DLS) measurements of small nanoparticles (NPs) (green), large NPs (red), negative control (mixture without SrtA, blue), and SrtA reaction product (black). (**b**,**c**) Transmission electron microscopy (TEM) images for NP–NP aggregates after SrtA reaction (**b**) and negative control without sortase (**c**).

In another ligation experiment, silica nanoparticles of 200 nm (functionalized with LPETG recognition motif) and 60 nm (with GGGGG peptide sequence) diameter were linked by SML. The unmodified small and large NPs were detected in DLS measurements with diameters in agreement to their size observed with TEM (Figure S2). The negative control sample with peptide-functionalized NPs showed a simple mixture of these two sizes of nanoparticles. In contrast, after SrtA reaction, no single nanoparticles were detected anymore. Instead, larger aggregates of different sizes were detected due to the linkages between large and small NPs (Figure 2a). TEM images showed that the large aggregates as well and most of the large nanoparticles are surrounded by small nanoparticles (Figure 2b). The negative control without SrtA also contained aggregates, but these were smaller and probably formed during drying of the sample (Figure 2c). However, due to unavoidable aggregation of NPs during sample preparation, a fully convincing conclusion is not possible with TEM. As the NP surfaces contain numerous peptides, the stoichiometry of the formed aggregates is not controllable and these structures were therefore not studied in more detail.

### 3.3. Formation of Block Copolymer via SML

**Figure 3:**
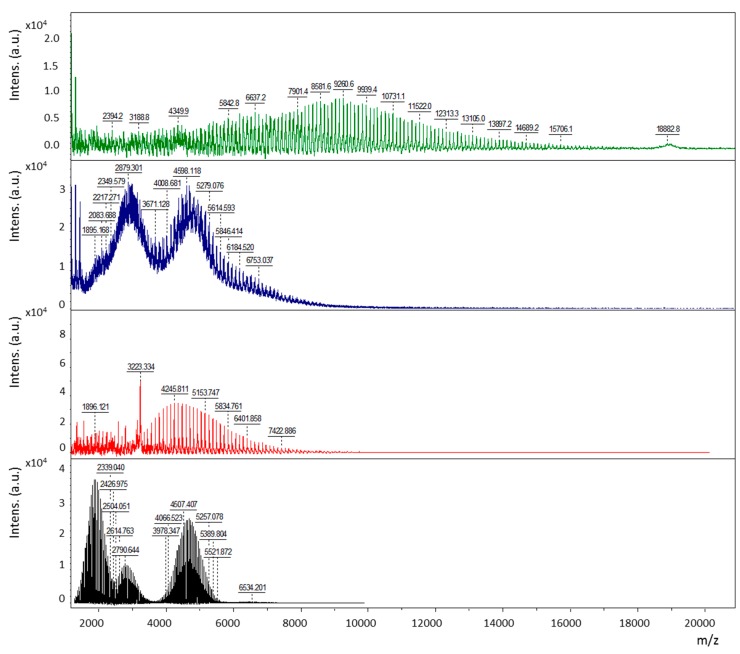
MALDI-ToF mass spectra of poly(ethylene glycol) (PEG)-peptide 3 (black), poly(*N*-isopropyl acrylamide) (PNIPAM)-peptide 4 (red), control without sortase (blue), and the product after the SrtA reaction (green).

Finally, as third linkage system, the formation of a block copolymer by SrtA catalysis was studied. The same polymers as before, PEG functionalized with oligoglycine motif containing peptide and PNIPAM with an LPETG motif containing peptide, were utilized. The polymer–peptide conjugates were then covalently linked by SrtA at 28 °C below the cloud point of aqueous PNIPAM solutions (ca. 32 °C). MALDI-ToF mass spectrometry proved the successful formation of the desired reaction product. As a result of the SrtA reaction, there is not only a shift to higher *m*/*z* values but also a much broader molecular weight distribution as expected for a block copolymer (Figure 3, green spectrum). The mass spectrum of a control experiment consisting of the reaction partners but omitting SrtA only revealed a mixture of the two polymer–peptide starting materials together with residual unmodified polymers (Figure 3, blue spectrum). A more detailed MS analysis of polymer starting materials, polymer–peptide conjugates and PNIPAM–peptide–PEG reaction product is presented in the Supporting Information (Figures S5–S11 and Tables S1–S4).

**Figure 4:**
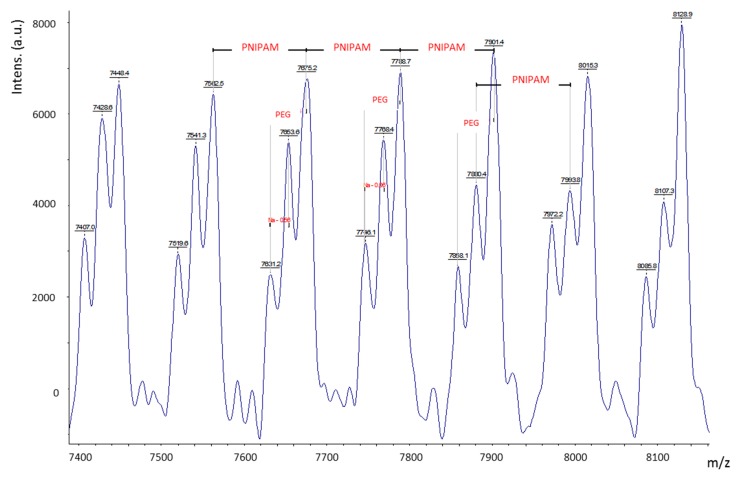
Section of the MALDI-ToF mass spectrum of the sortase A linked reaction product showing the presence of both poly(ethylene glycol) (PEG) and poly(*N*-isopropyl acrylamide) (PNIPAM) repeating units in the molecular ion series.

Figure 4 shows an excerpt of the mass spectrum of the block copolymer reaction product. Apart from the shift to higher molecular weight, the presence of multiple repeating units of both PEG and PNIPAM clearly reveals the linkage of the two polymer blocks. The unambiguous assignment of *m*/*z* values to defined ion species with the Polymerix software tool was not possible due to the complexity of the molecular architecture, but the successful linkage by sortase A is undoubtedly proven.

## 4. Discussion

NP–peptide and polymer–peptide conjugates were synthesized in order to demonstrate the linkage of these artificial building blocks by the enzyme sortase A. NP–polymer hybrids, NP–NP, and polymer–polymer structures were generated by sortase-mediated ligation and the product formation shown by transmission electron microscopy, MALDI-ToF mass spectrometry and dynamic light scatting. While we only addressed the shifting of the equilibrium of the sortase reaction by adding an excess of one building block up to now, to favor product formation can be achieved in the next years by many known techniques, e.g., removal of byproduct from the equilibrium under dialysis conditions or by being a poor nucleophile [3]. Interestingly, steric hindrance is a negligible factor for the investigated systems, since only few amino acids (3 to 13) were used as spacer to enable motif presentations for SrtA catalyzed ligation. In detail, spacers composed of three and four amino acids proved to be sufficient to successfully link two polymer blocks and a 13 amino acid spacer was used to immobilize peptides on bulky nanoparticles. The influence of the spacer length on the reaction efficiency, especially if the spacers can be shortened without compromising product formation, needs to be investigated in future.

For the proof of concept, the studied systems were kept as simple as possible by choosing easily accessible silica NPs and commercially available polymers although the approach also has distinct limitations. The purification of polymer–peptide conjugates was impossible and resulted in a mixture containing unmodified polymer. Future work will focus on more elaborate systems with similar building blocks. We are currently working on the synthesis of polymer–peptide building blocks that avoid a mixture of starting materials.

By demonstrating the linkage of exemplarily chosen synthetic structures, we developed a new strategy for the linkage of artificial building blocks by the enzyme sortase A. The validated linking principles can be transferred to all kinds of polymers and particles by attaching LPETG or GGGGG sequences. Therefore, the focus of this manuscript was on the proof of concept of the linkage strategy and characterization of the formed synthetic structures. More detailed analysis on general applicability, kinetics, yield, and scalability of the reactions will be performed in the future.

Although this proof of concept only shows examples that can be also synthesized by exclusively chemical techniques, a toolbox of such building blocks will enable the future formation of new materials and pave the way for the application of enzymes in materials science. In addition to nanoparticle systems and block copolymers, this also includes combination with protein-based building blocks to form hybrid materials. Hence, sortase could become an enzymatic tool that complements established chemical linking technologies [44] and provides specific peptide motifs that are orthogonal to all existing chemical functional groups. Compared to SML, other enzymatic ligation technologies suffer from broad specificity of recognition motifs or availability of enzymes as in the case of butelase [42,47]. In contrast, sortagging is already established and SrtA is even commercially available nowadays.

The presented results therefore not only prove the concept but also provide the strategy that SrtA can catalyze the covalent linkage of purely artificial chemical structures to form hybrid materials. This could become a very powerful alternative method to the current established chemical ligation. It is obvious that the use of expensive peptides does not allow for the synthesis of products in large scale, but it is a promising strategy to reach compounds where traditional synthetic chemistry approaches are limited.

## Supplementary Materials

The following are available online at http://www.mdpi.com/2073-4360/10/2/151/s1, Figure S1: Characterization of SrtA. (A) SDS-PAGE after SrtA purification. (B) MALDI-ToF mass spectrum of SrtA, Figure S2: TEM images of (unmodified) 60 nm and 200 nm SiO_2_ nanoparticles, Figure S3: Raman spectra of unmodified SiO_2_ NPs (black), MPS (red) and MPS-functionalized NPs (blue). The vibration bands at 1640 cm^−1^ (C=C) and 1724 cm^−1^ (C=O) show the successful surface modification, Figure S4: Raman spectra of C=C-functionalized NPs (black), Peptide 1 (red), and NP-Peptide hybrid materials (blue). The disappearance of the stretching vibration band of SH at 2571 cm^−1^, the appearance of a characteristic phenylalanine band at 1003 cm^−1^ and the appearance of the aromatic stretching vibration at 3061 cm^−1^ show the linkage of the peptide, Figure S5: Assignment of PEGMA spectral features to the homopolymer series S1–S3 specified in Table S1 (Polymerix Software, Sierra Analytics), Figure S6: Assignment of PNIPAM spectral features to the homopolymer series S1–S3 specified in Table S2 (Polymerix Software, Sierra Analytics), Figure S7: Assignment of PEG-peptide 3 conjugate spectral features to the homopolymer series S1–S8 specified in Table S3 (Polymerix Software, Sierra Analytics), Figure S8: Assignment of PNIPAM-peptide 4 spectral features to homopolymer series S1–S4 as specified in Table S4 (Polymerix Software, Sierra Analytics), Figure S9: MALDI-ToF mass spectra of the sortase A linked polymer-peptide conjugates (green) and their PEG-peptide 3 conjugate (black) and PNIPAM-peptide 4 (red) precursors without SrtA reaction, Figure S10: Section of the MALDI-ToF mass spectrum of the sortase A linked reaction product showing the presence of both PEG and PNIPAM repeating units in the molecular ion series, Figure S11: Structure of sortase A linked PNIPAM-peptide-PEG conjugates, Figure S12: Transmittance curves of the samples shown in Figure 1a,b: product from SrtA reaction 1 (black), negative control 2 (red, without polymer) and negative control 4 (blue, without SrtA). The measurements were done using SPECORD 210 UV-Vis spectrmeter from Analytik Jena. The concentration of all samples was 0.5 mg/mL. The wavelength was in the normal visible light range, 350–800 nm. The average transmittance of the samples was calculated through integration: SrtA reaction (1) 69.9%, negative control (2) 62.2%, negative control (4) 65.0%, Figure S13: FESEM images of 200 nm SiO_2_ NPs after linkage of PEG. The NPs are surrounded by a thin, water soluble polymer layer, Table S1: Assignment of PEGMA spectral features to homopolymer series S1–S3 (Polymerix Software, Sierra Analytics), Table S2: Assignment of PNIPAM spectral features to homopolymer series S1–S3 (Polymerix Software, Sierra Analytics), Table S3: Assignment of PEG-peptide 3 conjugate spectral features to homopolymer series S1–S8 (Polymerix Software, Sierra Analytics), Table S4: Assignment of PNIPAM-peptide 4 conjugate spectral features to homopolymer series S1–S4 (Polymerix Software, Sierra Analytics), Scheme S1: Overview of synthesis and surface modification of SiO_2_ NPs. First, the nanoparticles were synthesized by sol-gel method, then functionalized with a C=C coating through reaction with MPS and finally a peptide linked via Michael-type addition.
