# Supplementary material for: Sortase-Mediated Ligation of Purely Artificial Building Blocks
Source: Polymers (Basel). 2018 Feb 6;10(2):151. doi: 10.3390/polym10020151 (PMC6414994; doi:10.3390/polym10020151)
Supplement: Supplementary file 1 [file polymers-10-00151-s001.docx]

Supplementary Material

Sortase-catalyzed linkage of purely artificial building blocks

Xiaolin Dai, Diana M. Mate, Ulrich Glebe, Tayebeh Mirzaei Garakani, Andrea Körner, Ulrich Schwaneberg* and Alexander Böker*

Table of Contents

Sequences of the peptides used 2

Sortase A 2

Synthesis, characterization and surface functionalization of SiO_2_ NPs 3

MALDI-ToF MS characterization of polymer starting materials, polymer-peptide

conjugates, and sortase-linked product 5

Characterization of NP-polymer hybrids after SrtA reaction 12

**Sequences of the peptides used**

Peptide 1 linked to 200 nm NPs:

H-Cys-Ile-Arg-His-Met-Gly-Trp-Phe-Trp-Pro-Leu-Arg-Glu-Phe-Leu-Pro-Glu-Thr-Gly-OH

Peptide 2 linked to 60 nm NPs:

H-Gly-Gly-Gly-Gly-Gly-Phe-Glu-Arg-Leu-Pro-Trp-Phe-Trp-Gly-Met-His-Arg-Ile-Cys-OH

Peptide 3 linked to PEG:

H-Gly-Gly-Gly-Gly-Gly-Trp-Phe-Trp-Cys-OH

Peptide 4 linked to PNIPAM:

H-Cys-Ile-Arg-His-Phe-Leu-Pro-Glu-Thr-Gly-OH

**Sortase A**


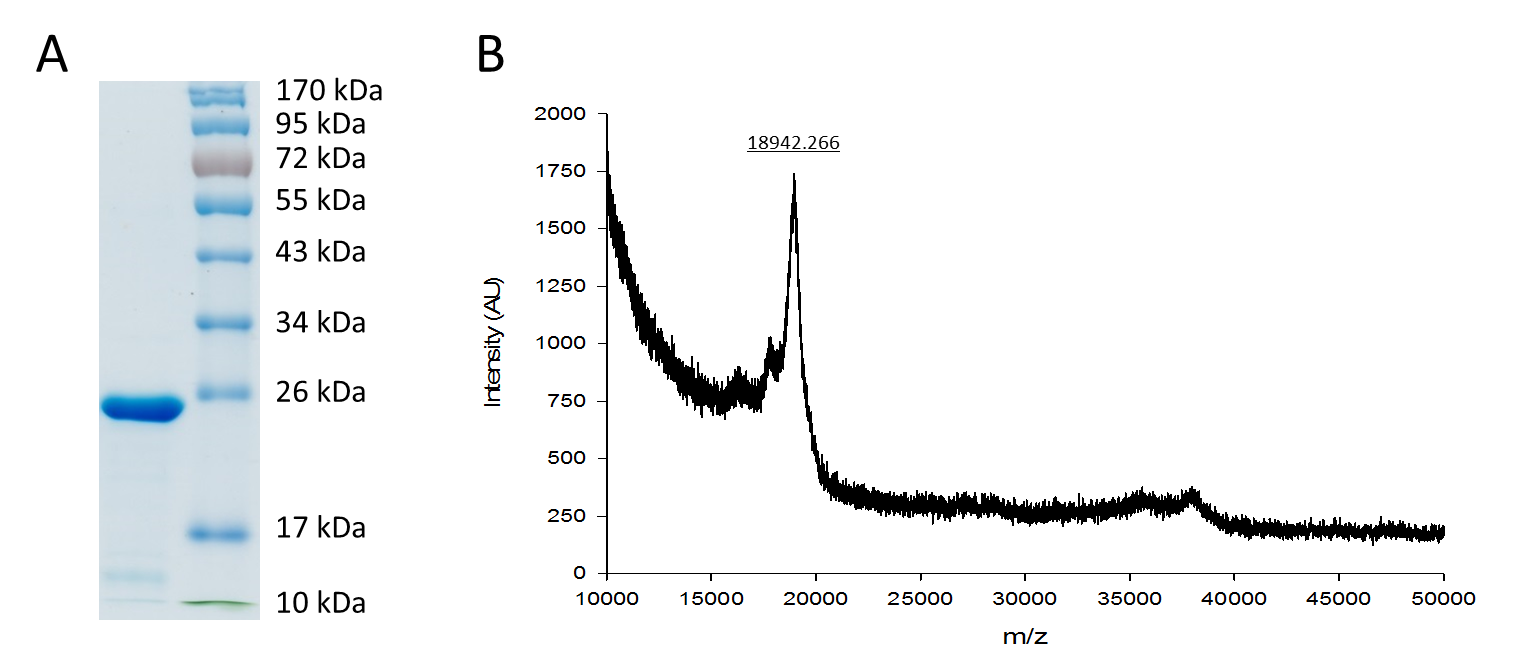


**Figure S1.** Characterization of SrtA. (A) SDS-PAGE after SrtA purification. (B) MALDI-ToF mass spectrum of SrtA.

**Synthesis, characterization and surface functionalization of SiO_2_ NPs**


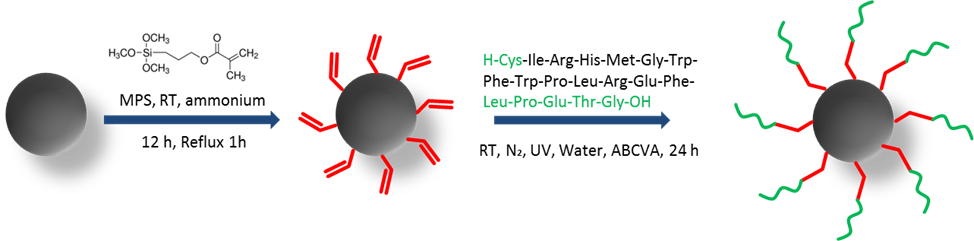


**Scheme S1.** Overview of synthesis and surface modification of SiO_2_ NPs. First, the nanoparticles were synthesized by sol-gel method, then functionalized with a C=C coating through reaction with MPS and finally a peptide linked via Michael-type addition.


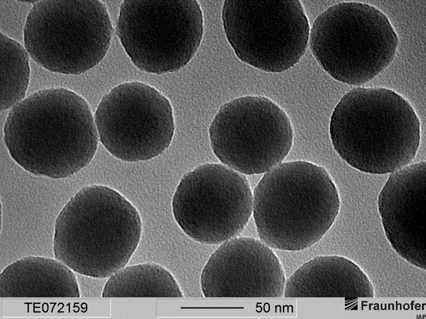

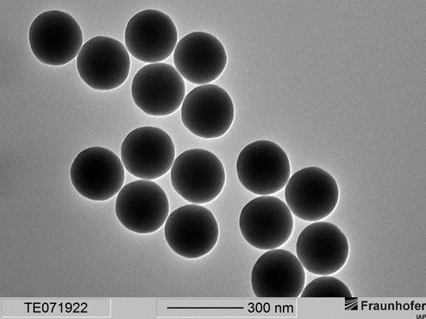


**Figure S2.** TEM images of (unmodified) 60 nm and 200 nm SiO_2_ nanoparticles.

**Figure S3.** Raman spectra of unmodified SiO_2_ NPs (black), MPS (red) and MPS-functionalized NPs (blue). The vibration bands at 1640 cm^-1^ (C=C) and 1724 cm^‑1^ (C=O) show the successful surface modification.

**Figure S4.** Raman spectra of C=C-functionalized NPs (black), Peptide 1 (red), and NP-Peptide hybrid materials (blue). The disappearance of the stretching vibration band of SH at 2571 cm^-1^, the appearance of a characteristic phenylalanine band at 1003 cm^-1^ and the appearance of the aromatic stretching vibration at 3061 cm^-1^ show the linkage of the peptide.

**MALDI-ToF MS characterization of polymer starting materials, polymer-peptide conjugates, and sortase-linked product**

Poly(ethylene glycol) methyl ether acrylate (PEGMA)

**Figure S5.** Assignment of PEGMA spectral features to the homopolymer series S1-S3 specified in Table S1 (Polymerix Software, Sierra Analytics).

The mass spectrum of PEGMA (S1 and S3) shows a bimodal distribution with a total/average M_n_ of 2713.8 Da, an average M_n_ of 1927.3 Da for the partial series with the lower and an average M_n_ of 3973.7 Da for the one with the higher masses (Table S1).

**Table S1.** Assignment of PEGMA spectral features to homopolymer series S1-S3 (Polymerix Software, Sierra Analytics)

| Series | M_n_ | M_w_ | M_z_ | PD | DP_n_ | DP_w_ | DP_z_ | Percent Series | Percent  Spectrum |
| --- | --- | --- | --- | --- | --- | --- | --- | --- | --- |
| Total/Average | 2667.218 | 3069.459 | 3471.892 | 1.151 | 58.825 | 67.962 | 77.103 | 100 | 95.67 |
| S1 | 2713.812 | 3117.959 | 3500.632 | 1.149 | 59.687 | 68.866 | 77.558 | 80.61 | 77.12 |
| S2 | 2309.982 | 2687.705 | 3181.627 | 1.164 | 51.741 | 60.32 | 71.539 | 16.06 | 15.37 |
| S3 | 3263.082 | 3737.526 | 4176.951 | 1.145 | 72.163 | 82.939 | 92.92 | 3.33 | 3.18 |

| Series Label | Alpha  End Group | Repeat | Omega  End Group | Charge State | Adduct | Adduct Charge | Formula |
| --- | --- | --- | --- | --- | --- | --- | --- |
| S1 | CH_3_ | C_2_H_4_O | C_3_H_3_O_2_ | 1 | Na | 1 | CH_3_ [C_2_H_4_O]n C_3_H_3_O_2_ + Na |
| S2 | CH_3_ | C_2_H_4_O | OH | 1 | Na | 1 | CH_3_ [C_2_H_4_O]n OH + Na |
| S3 | CH_3_ | C_2_H_4_O | C_3_H_3_O_2_ | 1 | K | 1 | CH_3_ [C_2_H_4_O]n C_3_H_3_O_2_+ K |

Poly(*N*-isopropylacrylamide) (PNIPAM) with maleimide chain-end functionalization

**Figure S6.** Assignment of PNIPAM spectral features to the homopolymer series S1-S3 specified in Table S2 (Polymerix Software, Sierra Analytics).

The assignment of distinct ion series to the spectrum of the PNIPAM chain-end functionalized with maleimide groups was made difficult by the lack of information on the alpha-end group by the polymer provider. Common initiators for the synthesis of PNIPAM via ATRP are α-bromoisobutyric acid (BIBA, C_4_H_7_O_2_) or azobisisobutyronitrile (AIBN, C_4_H_6_N); the homolytical cleavage of AIBN, e.g., produces two 2-cyanoprop-2-yl radicals which form the corresponding alpha-end group. The optimal assignment to the spectral features of the maleimide chain-end functionalized PNIPAM was in this case, however, obtained with a simple hydrogen atom forming the alpha-end group (Table S2).

**Table S2.** Assignment of PNIPAM spectral features to homopolymer series S1-S3 (Polymerix Software, Sierra Analytics)

| Series Label | M_n_ | M_w_ | M_z_ | PD | DP_n_ | DP_w_ | DP_z_ | Percent  Series | Percent  Spectrum |
| --- | --- | --- | --- | --- | --- | --- | --- | --- | --- |
| Total/Average | 2940.566 | 3799.202 | 4713.751 | 1.294 | 24.048 | 31.641 | 39.729 | 100 | 29.18 |
| S1 | 2806.555 | 3636.466 | 4550.281 | 1.296 | 22.678 | 30.017 | 38.097 | 31.31 | 9.13 |
| S2 | 3233.803 | 4104.834 | 4935.246 | 1.269 | 26.898 | 34.601 | 41.944 | 41.95 | 12.24 |
| S3 | 2637.568 | 3510.388 | 4557.735 | 1.331 | 21.183 | 28.902 | 38.163 | 26.75 | 7.8 |

| Series Label | Alpha-End Group | Repeat | Omega- End Group | Charge | Adduct | Formula |
| --- | --- | --- | --- | --- | --- | --- |
| S1 | H | C_6_H_11_ON | SCH_2_CH_2_NH(CO)CH_2_CH_2_ CH_2_(C_4_H_2_NO_2_) | 1 | Na | H [C_6_H_11_ON]_n_ SCH_2_CH_2_NH(CO)CH_2_CH_2_ CH_2_(C_4_H_2_NO_2_) + Na |
| S2 | C_4_H_7_O_2_ | C_6_H_11_ON | SCH_2_CH_2_COOH | 1 | Na | C_4_H_7_O_2_ [C_6_H_11_ON]n SCH_2_CH_2_COOH  + Na |
| S3 | H | C_6_H_11_ON | SCH_2_CH_2_NH(CO)CH_2_CH_2_ CH_2_(C_4_H_2_NO_2_) | 1 | Li | H [C_6_H_11_ON]_n_  SCH_2_CH_2_NH(CO)CH_2_CH_2_CH_2_(C_4_H_2_NO_2_)  + Li |

PEG-Peptide 3 conjugate

**Figure S7.** Assignment of PEG-peptide 3 conjugate spectral features to the homopolymer series S1-S8 specified in Table S3 (Polymerix Software, Sierra Analytics).

Four series S3-S6 (MH^+^, MNa^+^, MK^+^, and MNH_4_^+^ species), colored in blue, were obtained for PEG-peptide 3 conjugates. The bimodal distribution observed for the PEGMA starting material is maintained but accompanied with a mass shift to higher values due to the linkage of the peptide (MW = 926 g/mol). Residual PEGMA and PEG were detected as well (Table S3).

**Table S3.** Assignment of PEG-peptide 3 conjugate spectral features to homopolymer series S1-S8 (Polymerix Software, Sierra Analytics)

| Series Label | M_n_ | M_w_ | M_z_ | PD | DP_n_ | DP_w_ | DP_z_ | Percent Series | Percent  Spectrum |
| --- | --- | --- | --- | --- | --- | --- | --- | --- | --- |
| Total/Average | 3106.419 | 3485.768 | 3847.545 | 1.123 | 59.173 | 67.789 | 76.007 | 100 | 90.51 |
| S1 | 3194.554 | 3560.673 | 3917.722 | 1.115 | 70.606 | 78.922 | 87.032 | 6.05 | 5.47 |
| S2 | 3398.037 | 3731.395 | 4028.177 | 1.098 | 75.228 | 82.8 | 89.541 | 10.4 | 9.41 |
| S3 | 2686.168 | 3019.729 | 3400.209 | 1.124 | 38.04 | 45.617 | 54.259 | 16.27 | 14.72 |
| S4 | 3127.648 | 3478.705 | 3831.261 | 1.112 | 48.068 | 56.042 | 64.05 | 5.83 | 5.27 |
| S5 | 3390.837 | 3729.981 | 4031.661 | 1.1 | 54.046 | 61.749 | 68.602 | 9.96 | 9.01 |
| S6 | 3299.075 | 3703.732 | 4060.022 | 1.123 | 51.962 | 61.153 | 69.246 | 14.96 | 13.54 |
| S7 | 3029.715 | 3479.199 | 3892.599 | 1.148 | 68.089 | 78.298 | 87.688 | 24.57 | 22.23 |
| S8 | 3049.471 | 3409.363 | 3759.979 | 1.118 | 68.537 | 76.712 | 84.676 | 11.98 | 10.84 |

| Label | Alpha  End Group | Repeat | Omega  End Group | Charge State | Adduct | Formula |
| --- | --- | --- | --- | --- | --- | --- |
| S1 | CH_3_ | C_2_H_4_O | C_3_H_3_O_2_ | 1 | Na | CH_3_ [C_2_H_4_O]_n_ C_3_H_3_O_2_+ Na |
| S2 | CH_3_ | C_2_H_4_O | C_3_H_3_O_2_ | 1 | K | CH_3_ [C_2_H_4_O]_n_ C_3_H_3_O_2_+ K |
| S3 | CH_3_ | C_2_H_4_O | OOC-CH_2_-CH_2_-S-C_44_H_50_N_11_O_10_ | 1 | H | CH_3_ [C_2_H_4_O]_n_ OOC-CH_2_-CH_2_-S- C_44_H_50_N_11_O_10_ + H |
| S4 | CH_3_ | C_2_H_4_O | OOC-CH_2_-CH_2_-S-C_44_H_50_N_11_O_10_ | 1 | Na | CH_3_ [C_2_H_4_O]_n_ OOC-CH_2_-CH_2_-S- C_44_H_50_N_11_O_10_ + Na |
| S5 | CH_3_ | C_2_H_4_O | OOC-CH_2_-CH_2_-S-C_44_H_50_N_11_O_10_ | 1 | K | CH_3_ [C_2_H_4_O]_n_ OOC-CH_2_-CH_2_-S- C_44_H_50_N_11_O_10_ + K |
| S6 | CH_3_ | C_2_H_4_O | OOC-CH_2_-CH_2_-S-C_44_H_50_N_11_O_10_ | 1 | NH_4_ | CH_3_ [C_2_H_4_O]_n_ OOC-CH_2_-CH_2_-S- C_44_H_50_N_11_O_10_ + NH_4_ |
| S7 | CH_3_ | C_2_H_4_O | OH | 1 | Na | CH_3_ [C_2_H_4_O]_n_ OH + Na |
| S8 | CH_3_ | C_2_H_4_O | OH | 1 | K | CH_3_ [C_2_H_4_O]_n_ OH + K |

PNIPAM-Peptide 4 conjugate

**Figure S8.** Assignment of PNIPAM-peptide 4 spectral features to homopolymer series S1-S4 as specified in Table S4 (Polymerix Software, Sierra Analytics).

While the PNIPAM oligomers were detected as sodium ions (Table S2), the charge of the peptide conjugate is mainly provided by protonation, presumably of the peptide part, and to a lower extent by sodium adducts (S1, S2 in Table S4). The successful linkage of the peptide causing a shift of 1150 Da (peptide 4 with a MW = 1172 g/mol) is proven. Like for the PNIPAM starting material (Table S2) the optimal assignment to the spectral features is again obtained with a hydrogen atom forming the alpha-end group of the PNIPAM-peptide conjugate. To a lesser extent series were also assigned PNIPAM with BIBA and AIBN alpha-end groups.

**Table S4.** Assignment of PNIPAM-peptide 4 conjugate spectral features to homopolymer series S1-S4 (Polymerix Software, Sierra Analytics)

| Series Label | M_n_ | M_w_ | M_z_ | PD | DP_n_ | DP_w_ | DP_z_ | Percent Series | Percent Spectrum |
| --- | --- | --- | --- | --- | --- | --- | --- | --- | --- |
| Total/Average | 4363.871 | 5519.961 | 6820.906 | 1.265 | 25.79 | 36.014 | 47.518 | 100 | 37.11 |
| S1 | 4394.992 | 5390.436 | 6533.843 | 1.226 | 26.364 | 35.167 | 45.278 | 30.38 | 11.27 |
| S2 | 4351.796 | 5546.481 | 6855.732 | 1.275 | 25.982 | 36.547 | 48.124 | 25.37 | 9.41 |
| S3 | 4359.861 | 5591.238 | 6977.653 | 1.282 | 25.46 | 36.349 | 48.609 | 22.74 | 8.44 |
| S4 | 4338.396 | 5596.272 | 7019.565 | 1.29 | 25.103 | 36.226 | 48.812 | 21.51 | 7.98 |

| Series Label | Alpha  End Group | Repeat | Omega  End Group | Charge State | Adduct | Formula |
| --- | --- | --- | --- | --- | --- | --- |
| S1 | H | C_6_H_11_ON | SCH_2_CH_2_NH(CO)CH_2_CH_2_ CH_2_(C_4_H_2_NO_2_)C_52_H_81_N_15_O_14_S | 1 | H | H[C_6_H_11_ON]_n_SCH_2_CH_2_NH(CO)CH_2_CH_2_CH_2_ (C_4_H_2_NO_2_) C_52_H_81_N_15_O_14_S |
| S2 | H | C_6_H_11_ON | SCH_2_CH_2_NH(CO)CH_2_CH_2_ CH_2_(C_4_H_2_NO_2_)C_52_H_81_N_15_O_14_S | 1 | Na | H[C_6_H_11_ON]_n_SCH_2_CH_2_NH(CO)CH_2_CH_2_CH_2_ (C_4_H_2_NO_2_) C_52_H_81_N_15_O_14_S + Na |
| S3 | C_4_H_6_N | C_6_H_11_ON | SCH_2_CH_2_NH(CO)CH_2_CH_2_ CH_2_(C_4_H_2_NO_2_)C_52_H_81_N_15_O_14_S | 1 | H | C_4_H_6_N[C_6_H_11_ON]_n_SCH_2_CH_2_NH(CO)CH_2_CH_2_CH_2_ (C_4_H_2_NO_2_) C_52_H_81_N_15_O_14_S + H |
| S4 | C_4_H_7_O_2_ | C_6_H_11_ON | SCH_2_CH_2_NH(CO)CH_2_CH_2_ CH_2_(C_4_H_2_NO_2_)C_52_H_81_N_15_O_14_S | 1 | H | C_4_H_7_O_2_[C_6_H_11_ON]_n_SCH_2_CH_2_NH(CO)CH_2_CH_2_CH_2_ (C_4_H_2_NO_2_) C_52_H_81_N_15_O_14_S + H |

Sortase linked PNIPAM-peptide-PEG conjugates

**Figure S9.** MALDI-ToF mass spectra of the sortase A linked polymer-peptide conjugates (green) and their PEG-peptide 3 conjugate (black) and PNIPAM-peptide 4 (red) precursors without SrtA reaction.

The mass spectrum of the sortase linked polymer-peptide conjugates in Figure S9 reveals two main ion series with a shift to higher molecular weights when compared to the PEG‑peptide 3 and PNIPAM-peptide 4 conjugate precursors. The successful linkage by sortase A is further confirmed by the presence of the combined PEG and PNIPAM repeating units in the molecular ion series (excerpt of the mass spectrum in Figure 3). Due to the downright complex nature of the sortase A linked reaction product (Figure S11), the high number of possible polymer conjugates and resulting mass conflicts, an unambiguous assignment of m/z values to defined ion species with the Polymerix software tools is not possible.

**Figure S10.** Section of the MALDI-ToF mass spectrum of the sortase A linked reaction product showing the presence of both PEG and PNIPAM repeating units in the molecular ion series.

**Figure S11.** Structure of sortase A linked PNIPAM-peptide-PEG conjugates.

**Characterization of NP-polymer hybrids after SrtA reaction**


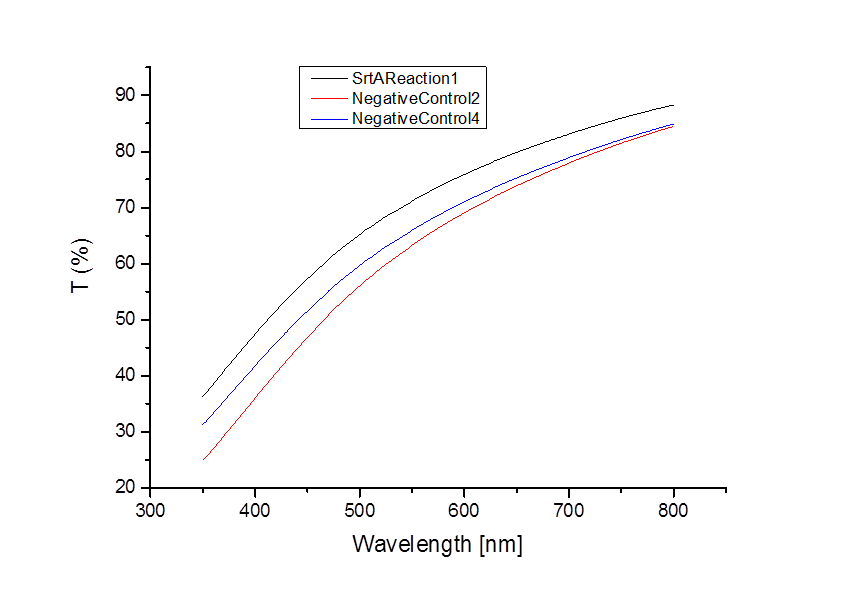


**Figure S12.** Transmittance curves of the samples shown in Figure 1 a and b: product from SrtA reaction 1 (black), negative control 2 (red, without polymer) and negative control 4 (blue, without SrtA). The measurements were done using SPECORD 210 UV-Vis spectrmeter from Analytik Jena. The concentration of all samples was 0.5 mg/mL. The wavelength was in the normal visible light range, 350-800 nm. The average transmittance of the samples was calculated through integration: SrtA reaction (1) 69.9 %, negative control (2) 62.2 %, negative control (4) 65.0 %.

**
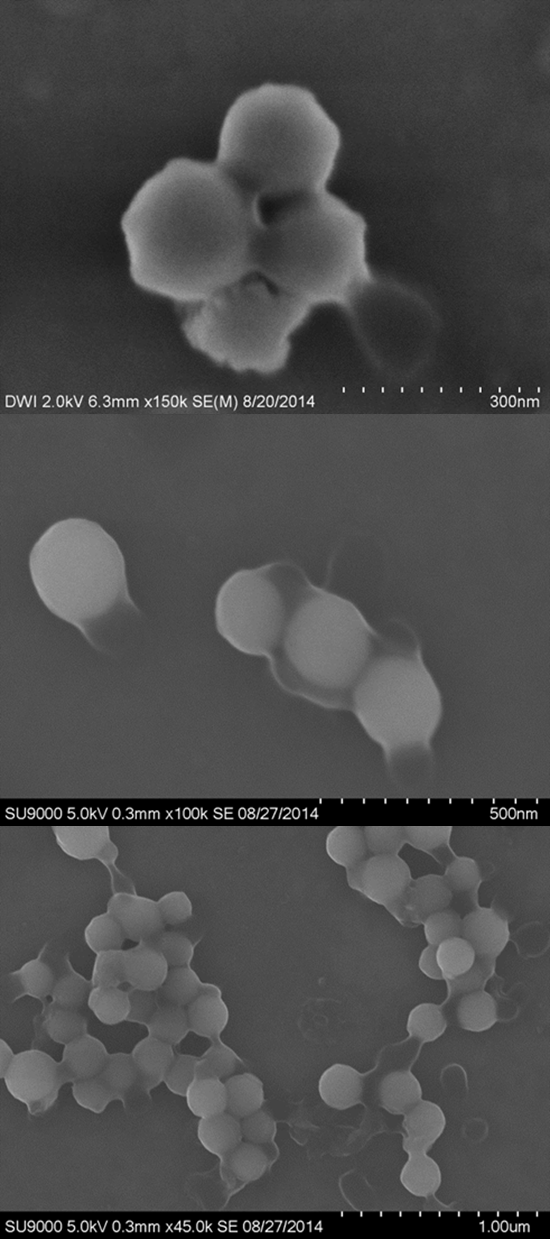
**

**Figure S13.** FESEM images of 200 nm SiO_2_ NPs after linkage of PEG. The NPs are surrounded by a thin, water soluble polymer layer.
